# Privacy-preserving dataset combination and Lasso regression for healthcare predictions

**DOI:** 10.1186/s12911-021-01582-y

**Published:** 2021-09-16

**Authors:** Marie Beth van Egmond, Gabriele Spini, Onno van der Galien, Arne IJpma, Thijs Veugen, Wessel Kraaij, Alex Sangers, Thomas Rooijakkers, Peter Langenkamp, Bart Kamphorst, Natasja van de L’Isle, Milena Kooij-Janic

**Affiliations:** 1 Unit ICT, TNO (Dutch Organization for Applied Scientific Research), The Hague, The Netherlands; 2 Leiden Institute of Advanced Computer Science, Leiden University, Leiden, The Netherlands; 3 Cryptology Research Group, Centrum Wiskunde and Informatica (CWI), Amsterdam, The Netherlands; 4 TMC Data Science, Eindhoven, The Netherlands; 5 Achmea, Zeist, The Netherlands; 6 Erasmus MC, Rotterdam, The Netherlands

**Keywords:** Secure multi-party computation, Privacy, Machine learning, Lasso regression

## Abstract

**Background:**

Recent developments in machine learning have shown its potential impact for clinical use such as risk prediction, prognosis, and treatment selection. However, relevant data are often scattered across different stakeholders and their use is regulated, e.g. by GDPR or HIPAA.

As a concrete use-case, hospital Erasmus MC and health insurance company Achmea have data on individuals in the city of Rotterdam, which would in theory enable them to train a regression model in order to identify high-impact lifestyle factors for heart failure. However, privacy and confidentiality concerns make it unfeasible to exchange these data.

**Methods:**

This article describes a solution where vertically-partitioned synthetic data of Achmea and of Erasmus MC are combined using Secure Multi-Party Computation. First, a secure inner join protocol takes place to securely determine the identifiers of the patients that are represented in both datasets. Then, a secure Lasso Regression model is trained on the securely combined data. The involved parties thus obtain the prediction model but no further information on the input data of the other parties.

**Results:**

We implement our secure solution and describe its performance and scalability: we can train a prediction model on two datasets with 5000 records each and a total of 30 features in less than one hour, with a minimal difference from the results of standard (non-secure) methods.

**Conclusions:**

This article shows that it is possible to combine datasets and train a Lasso regression model on this combination in a secure way. Such a solution thus further expands the potential of privacy-preserving data analysis in the medical domain.

## Background

Modern machine-learning techniques require large-scale and well-characterized datasets to achieve their full potential. In the medical domain, this requirement translates to a need to store medical patient data, and to combine information from different institutions, the Covid-19 outbreak being an example of a situation where this is deemed crucial [1, 2].

However, the collection, processing and exchange of personal data is a sensitive matter, and the risks coming from privacy violations are especially high for medical data. This has led to legal frameworks that regulate and restrict usage of personal (medical) data, the General Data Protection Regulation^1^ (GDPR), and the Health Insurance Portability and Accountability Act^2^ (HIPAA) being two prominent examples. These regulations mandate informed consent from patients in order to use the corresponding medical data; however, asking for consent for machine-learning purposes is often impractical, since it is a time-consuming process, and since contact with patients may have been lost since the moment of data collection.

This conflict between, on the one hand, the need to gather, combine and process large amounts of data for better machine-learning techniques, and on the other hand the need to minimize personal data usage for privacy protection, has lead to the development of several solutions for privacy-preserving data analysis. In particular, a collection of cryptographic techniques known as Secure Multi-Party Computation, or MPC for short, is being applied more and more in the medical domain. Intuitively, the goal of MPC is to allow several parties to compute the output of a certain function or computation, depending on private inputs of each party, without actually disclosing information on their inputs to each other.

In 2018, the Netherlands Organization for Applied Scientific Research (TNO), together with academic medical center Erasmus MC and health insurance company Achmea, started a project within the Horizon 2020 Programme called BigMedilytics^3^ to develop a secure algorithm to predict the number of hospitalization days for heart failure patients. Although the project does not use real patient data in its current phase, the MPC solution presented in this article is based on the following real-life use-case, which serves as a motivating example for the solution described in this article. In Rotterdam, a group of individuals took part in the “Rotterdam study” [3], a program by the Epidemiology department of Erasmus MC. Erasmus MC has collected data on the lifestyle of these patients, for example their exercising, smoking, and drinking behavior. Achmea, on the other hand, has claims data of its customers (including several participants of the Rotterdam study), which encompass different aspects, such as hospitalization days and healthcare usage outside of the hospital. Recent work has shown that using machine-learning models on medical data has the potential to predict survival of heart-failure patients [4]. The datasets of Achmea and Erasmus MC, once intersected and combined, could be used to train a prediction model that identifies high-impact lifestyle factors for heart failure, and thus, in turn, to recognize high-risk heart-failure patients.

However, privacy concerns mean that Erasmus MC and Achmea cannot simply share their data with each other to allow for a straightforward analysis. TNO has therefore developed and implemented the MPC-based Proof of Concept described in this article, which allows Erasmus MC and Achmea to securely train a prediction model without disclosing any personal medical information.

Before we present the details of our solution, we give an overview of the current landscape of privacy-preserving data analysis techniques, focusing on the medical domain, and on solutions which bear resemblance to ours. We will then discuss how our solution compares to these existing techniques.

### Previous and related work

#### Secure analysis of healthcare data

A straightforward approach for privacy-preserving data analytics consists of data anonymization and pseudonymization. More precisely, in the case of horizontally-partitioned data (i.e. when organizations hold the same type of data on different individuals), organizations may simply remove identifiers such as name, date of birth, or social security numbers, and share the data features with each other; in the case of vertically-partitioned data (i.e. when parties hold the different data on the same individuals), a similar result can be achieved by resorting to an external third party that gets access to all identifiers, replaces them with pseudonyms, and then ensures that the data features from all involved organizations are linked to each other. These methods thus ensure that only feature data are revealed, instead of identifiers. However, feature data can often uniquely identify an individual, especially if other, related data is acquired through public sources, as shown in several studies [5, 6]. Thus in practice, data anonymization and pseudonymization offer little guaranteed on the protection of the identity of individuals involved in collaborative data analysis.

A more sophisticated and popular approach consists of federated learning, where algorithms are trained on decentralized devices or servers, each possessing its own data, by only exchanging intermediate model coefficients with each other. Federated learning promises great potential to facilitate big data for medical application, in particular for international consortia [7].

An example of federated-learning architecture is provided by the Personal Health Train (PHT) [8], which core idea is to keep data at the original sources and to let the analytical tasks “visit” these data sources and perform data analysis tasks locally.

Both federated learning and the PHT work fairly straightforward for horizontally-partitioned data (where institutions hold the same type of data on different individuals), while vertically-partitioned data remains a challenge to be tackled.

Cryptographic solutions such as MPC typically overcome these limitations, but with an inherent overhead in terms of computation time and communication volume compared to non-cryptographic solutions, and typically have a lower technology readiness level. Specific applications in the medical domain cover a wide range, including, for instance, disclosure of case counts, while preserving confidentiality of healthcare providers [9], sharing insights on the effectiveness of HIV treatments, while preserving both privacy of involved patients and confidentiality of practitioners’ treatment choices [10]; privacy-preserving analysis of hospital workflows [11]; secure genome study [12]; and secure distributed logistic regression for medical data [13]. Compliance of MPC techniques with the GDPR has been discussed in [14].

We present in more details MPC solutions with a similar scope as ours in the following sub-section.

#### Cryptographic techniques for dataset combination and secure regression

A first challenge in secure distributed data analysis lies in the combination of different datasets: namely, different institutions hold, in general, data on different individuals, and a first challenge lies in determining which individuals lie both datasets, and retrieving their relevant features. Various work has been done on “secure set intersection” (also referred to as “private set intersection”) [15–18], where the different involved parties learn which individuals lie in all datasets, but it is guaranteed that no information on individuals outside the intersection will be revealed. To the best of our knowledge, however, no previous work has been published that describes a *secure inner join* solution, where individuals in the intersection are determined, but not revealed, and where the corresponding feature values are associated to each individual. Notice that a secure inner join is a fundamental step for realistic deployment of a secure data analysis solution, since the identity of individuals in the intersection of datasets is, in general, personal (and thus protected) data.

Concerning securely training a linear regression model on distributed data, a lot of work has been done on a variant of linear regression known as Ridge regression. In linear regression, one aims to find a “coefficient” vector, such that the linear combination of feature values with the coefficients yields roughly the value of another, “target” feature. Such a linear combination cannot, in general, be exactly equal to the target feature: Ridge regression is a relatively straightforward method that aims to find a coefficient vector that minimizes this gap, while also preventing the obtained coefficient models from being biased towards the training features values (and thus poorly predict the target feature for new data). Ridge regression is typically solved in either of two ways: by solving the normal equations, or by minimizing the objective function in a more general fashion, e.g. by application of the Gradient Descent algorithm. The privacy-preserving implementations [19–26] all train a Ridge regression model by solving the normal equations, which is in turn performed through matrix inversion. Privacy is often preserved by using homomorphic encryption techniques [20–23], yet there are also implementations that make of use of secret sharing [19], or of garbled circuits [24].

In contrast to the normal-equations approach, we chose for the secure Gradient Descent approach. The works of [27, 28] all present privacy-preserving Gradient Descent solutions to train Ridge regression models. In [28] and [27] the authors focus on vertically-partitioned data. Finally, the authors of [27] train a linear regression model via a variation of the standard Gradient Descent method, namely conjugate Gradient Descent.

The solution that we present in this article focuses on another linear regression method called Lasso; to the best of our knowledge, no previous work has been published on secure Lasso regression. Lasso is similar to Ridge in that it tries to minimize the gap between the target feature and the linear combination of the other features with the coefficient vector, but it also discards features of little impact on the target feature by pushing the corresponding coefficient to zero. This means that once the model has been (securely) trained, less data are needed to evaluate the model. This is a very desirable property for a healthcare-prediction scenario, and in particular for the identification of high-impact factors for heart failure, as described at the beginning of this section: gathering and using only the data that is strictly necessary to apply the model is important to comply with privacy regulations and their data-minimization requirements. In [4] it is even shown that for the prediction of the survival of heart-failure patients, training a model on two features alone can yield more accurate predictions than those made using all available features.

### Our contributions

We present a solution for (1) computing a secure inner join of two datasets and, (2) securely training a Lasso regression model on the obtained (encrypted) data. To the best of our knowledge, both these contributions are novel.

Both components of our solution are essential: the secure inner join ensures that individuals in the overlap of the two datasets can be determined (but not revealed) together with their feature values, and the Lasso regression allows for minimizing the number of features that have an impact on the model, thus meeting the proportionality and data-minimization requirements for subsequent non-encrypted application of the model to identify high-risk patients.

In both components, we assume that a third, “helper” party joins the computation. The helper party does not supply any input and does not learn the input data of the other parties nor the outcome of the model, but its inclusion allows for a very efficient design. Namely, we are able to use efficient techniques such as hashing for the secure inner join, as opposed to the expensive polynomial-evaluation techniques typically required in a two-party setting; for what concerns the secure Lasso regression, we can make use of the MPyC framework, which requires at least three parties to guarantee security.

Our solution is tailored to the heart-failure use-case described above, and involves Achmea and Eramus MC as data parties and healthcare information intermediation company ZorgTTP as helper party. We installed our solution on a test infrastructure of the three involved parties, generated artificial data, and tested the performance in terms of quality of the obtained model and efficiency. Both aspects are fully satisfactory, the secure solution showing a difference in objectives of 0.004 with a standard, non-secure solution (in scikit-learn), and requiring less than one hour to compute the inner join and Lasso coefficients of two datasets, consisting of 5000 records each, and 30 features in total.

### Outline

The rest of the article is organized as follows. The "Methods" section is divided into two parts: the first one (section "Description of the desired functionality") illustrates the functionality that we aim to achieve (inner join and Lasso regression), but without taking security and privacy considerations into account. These are discussed in the following section "Description of the secure solution", which shows how our solution securely implements the functionality of section "Description of the desired functionality". The "Results" section discusses what our solution achieves in terms of security (section "Security results"), efficiency (section "Running time"), and quality of the obtained regression model (section "Performance and accuracy results"). We discuss the impact and possible improvements of our work in section "Discussion", and we end with the conclusions in section "Conclusions".

## Methods

### Description of the desired functionality

We first discuss the details of the functionality that we aim to realize. Privacy and security aspects are not considered here, and will instead be discussed in section "Description of the desired functionality", following the same structure as the current section.

#### Description of the setting and data formatting

We begin with the general set-up and a description of the format of the input data. In our setting, two data-providing parties are involved: a healthcare insurance company, *Achmea* (often shortened to *AC*), and a university hospital, *Erasmus MC* (which will often be shortened to *EMC*). We assume that each party owns a dataset where several *features* of various customers/patients are contained. Each row in the dataset corresponds to a customer or patient, and we refer to it as a *record.* Specifically, we denote the dataset of Achmea, and its element, as in Table 1, and we denote by $A$ its set of identifiers $\{a_1, a_2, \ldots \}$.

**Table 1:** AC dataset

Identifier	Feature $\alpha ^{\left( 1 \right) }$	...	Feature $\alpha ^{\left( \ell \right) }$
$a_1$	$\alpha _1^{\left( 1 \right) }$	...	$\alpha _1^{\left( \ell \right) }$
$a_2$	$\alpha _2^{\left( 1 \right) }$	...	$\alpha _2^{\left( \ell \right) }$
$\vdots$	$\vdots$	$\vdots$	$\vdots$

The dataset of Erasmus MC, on the other hand, is depicted as in Table 2, and we denote by $B$ the set of identifiers $\{b_1,b_2, \ldots \}$.

**Table 2:** EMC dataset

Identifier	Feature $\beta ^{\left( 1 \right) }$	...	Feature $\beta ^{\left( m \right) }$
$b_1$	$\beta _1^{\left( 1 \right) }$	...	$\beta _1^{\left( m \right) }$
$b_2$	$\beta _2^{\left( 1 \right) }$	...	$\beta _2^{\left( m \right) }$
$\vdots$	$\vdots$	$\vdots$	$\vdots$

Before discussing the properties of identifiers and features, we stress the fact that the research described in this article did *not* use any actual identifiers or features corresponding to existing individuals. For the running time, accuracy and performance experiments, synthetic data was created or existing public data sets were used. More details can be foud in "Results" section.

It is assumed that identifiers in $A$ and in $B$ are of the same type; for simplicity, one may think of them as the social security number of a customer/patient. In particular, if $a_i$ and $b_j$ refer to the same person, then $a_i=b_j$. Notice that we are actually interested in the intersection of $A$ and $B$, as we want to train a regression algorithm on all features.

For what concerns the features, both $\alpha ^{\left( i \right) }$ and $\beta ^{\left( j \right) }$ are assumed to be numerical or Boolean. One of the features serves as a *target:* intuitively, we aim to predict its value as a function of the other feature values. We formalize this intuitive goal in the following sub-sections.

#### Inner join of the data

In order to find a correlation among different features, a first necessary step is to identify which features belong to the same customer/patient. Namely, not every person of Achmea is necessarily present in the database of Erasmus MC (as not all customers of AC took part in the social and behavioral study of EMC), and vice versa. In mathematical terms, and in the notation defined above, $A\ne B$ (in general).

Therefore, the two parties need to a) compute $A\cap B$ (i.e. identify which persons are represented in both databases), b) ensure that $\alpha _i^{\left( \cdot \right) }$ and $\beta _j^{\left( \cdot \right) }$ are identified for all *i* and *j* such that $a_i=b_j\in A\cap B$ (i.e. assign to each identifier in the intersection the corresponding features). In Tables 3 and 4, an example of the aimed result of this intersection is shown, inspired by the heart-failure use-case presented in the background section.

**Table 3:** Achmea and Erasmus MC example datasets, respectively

Identifier	Hospitalization days	Identifier	Hours of exercise per week
000000	10	000000	0
111111	5	111111	2
555555	8	777777	1
777777	9	999999	3

**Table 4:** Inner Join example

Identifier	Hospitalization days	Hours of exercise per week
000000	10	0
111111	5	2
777777	9	1

More abstractly, Table 5 would therefore be obtained, using the notation of Tables 1 and 2.

**Table 5:** Inner-join dataset

Identifier	Feature $\alpha ^{\left( 1 \right) }$	...	Feature $\alpha ^{\left( \ell \right) }$	Feature $\beta ^{\left( 1 \right) }$	...	Feature $\beta ^{\left( m \right) }$
$a_{i_1}=b_{j_1}$	$\alpha _{i_1}^{\left( 1 \right) }$	...	$\alpha _{i_1}^{\left( \ell \right) }$	$\beta _{j_1}^{\left( 1 \right) }$	...	$\beta _{j_1}^{\left( m \right) }$
$a_{i_2}=b_{j_2}$	$\alpha _{i_2}^{\left( 1 \right) }$	...	$\alpha _{i_2}^{\left( \ell \right) }$	$\beta _{j_2}^{\left( 1 \right) }$	...	$\beta _{j_2}^{\left( m \right) }$
$\vdots$	$\vdots$	$\vdots$	$\vdots$	$\vdots$	$\vdots$	$\vdots$

This type of operation is commonly referred to as *Inner Join* in the field of database management [29].

The next step is to train a regression algorithm on the data contained in Table 5. We remark that, at this point, the identifier column is no longer necessary, and indeed will play no role in the regression step.

#### Lasso regression algorithm

Given Table 5, we are now interested in finding a way of expressing a given feature (the number of hospitalization days) as a linear combination of the other features, or as an approximation of such a linear combination. This is accomplished by training a linear regression model on Table 5. In this subsection, we discuss the details of this process.

A linear regression problem can be informally expressed by the following question: for a known matrix $\mathbf {X}\in \mathbb {R}^{n \times m}$, where *n* is the number of records and *m* is the number of features, and *target* vector $\mathbf {y}\in \mathbb {R}^{n \times 1}$, can we find a *weight vector* $\mathbf {w}$ such that the equality $\mathbf {X}\mathbf {w}= \mathbf {y}$ is satisfied? In general the system is over-determined and there exists no solution. Instead, one aims to find $\mathbf {w}$, such that some function of the approximation error vector $\mathbf {X}\mathbf {w}-\mathbf {y}$ (and possibly some other arguments) is minimized.

The straightforward form of this problem focuses on minimizing the $\ell ^2$-norm $\Vert \mathbf {X}\mathbf {w}- \mathbf {y}\Vert _2^2$, where $\Vert \mathbf {x}\Vert _2^2:=\sum _i x_i^2$; this is known as (ordinary) least squares linear regression (OLS) [30]. Typically, a so-called regularization term is added to this target value; for instance, Ridge regression [31, 32] uses the $\ell ^2$-norm of the weight vector, and therefore tries to minimize the value $\Vert \mathbf {X}\mathbf {w}- \mathbf {y}\Vert _2^2 + \lambda \Vert \mathbf {w}\Vert _2^2$, for a fixed constant $\lambda >0$. The goal of such a regularization term is to ensure that the weight vector has small values, thereby making the overall model more manageable and reducing the risk of overfitting (i.e. reducing the risk that the model is too tailored to the data $\mathbf {X},\mathbf {y}$ and poorly predicts values based on new data).

We choose instead for Lasso (Least Absolute Shrinkage and Selection Operator) [33, 34], which automatically discards features of little impact on the target vector. This is a desirable feature for the use-case described in the Background section, as it is important to focus on factors that have the greatest impact on hospitalization days, in order to minimize data collection for subsequent usage of the obtained model. Furthermore, reducing the number of used features results in a more easily explainable model, thereby increasing the acceptance by end-users. Lasso tries to minimize the following objective function:

1 $$\begin{aligned} F(\mathbf {w}) = \frac{1}{n} \Vert \mathbf {X}\mathbf {w}-\mathbf {y}\Vert _2^2 + \lambda \Vert \mathbf {w}\Vert _1, \end{aligned}$$

where $\Vert \mathbf {w}\Vert _1 = \sum _{i=1}^n |w_i|$, and where $\lambda >0$ is a user-chosen parameter known as *regularization parameter*. Note that this method reduces to OLS, if $\lambda$ is set to zero. In our set-up we use a proximal gradient descent algorithm to minimize the objective.

#### Gradient descent approach

Gradient Descent (GD) is a general optimization algorithm that finds a local minimum of an objective function. The algorithm takes repeated steps in the opposite direction of the (approximate) gradient of the objective function at the current point. In that way it moves to the direction of steepest descent. GD is a building block for many different models, including Ridge Regression and Support Vector Machine. The GD algorithm is described in Algorithm 1. We will now describe the parameters and functions in this algorithm.

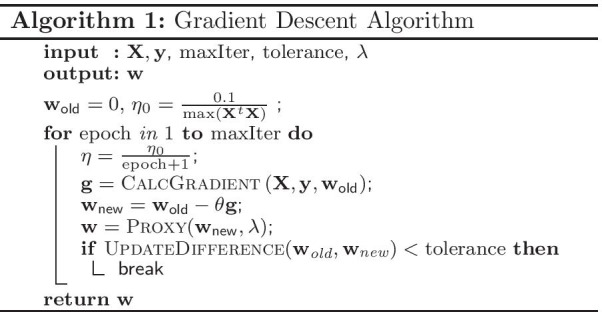



*Stopping criteria.* The algorithm can stop for two reasons: either because it has reached the limit of iterations set by $\text {maxIter}$, or when the model has been trained “sufficiently”. The latter factor can be quantified by measuring the relative or absolute change in the objective function and comparing this change with a pre-set treshold. Since the secure evaluation of an objective function is computationally intensive, we compare with a treshold the following value, known as *update difference:*

2 $$\begin{aligned} \textsc {UpdateDifference}(\mathbf {w}_{\text {new}},\mathbf {w}_{\text {old}}) = \frac{||\mathbf {w}_{\text {new}}-\mathbf {w}_{\text {old}}||_2^2}{||\mathbf {w}_{\text {old}}||_2^2}. \end{aligned}$$

The model is then said to be sufficiently trained when this value is smaller than a pre-set value known as *tolerance.*

$\textsc {CalcGradient}$*and*$\textsc {Proxy}$ In the GD algorithm, we repeatedly calculate the gradient $\textsc {CalcGradient}$. Because of the $\ell ^1$-norm in (1), the objective function for Lasso is non-differentiable. We therefore use the technique of proximal gradient descent, that can optimize a function that is not entirely differentiable. We therefore first compute a gradient function over the first part of the objective function in (1),

3 $$\begin{aligned} \textsc {CalcGradient}(\mathbf {w}) := \frac{2}{n} \mathbf {X}^T(\mathbf {X}\mathbf {w}-\mathbf {y}), \end{aligned}$$

then approximate the gradient of the second part of (1) by applying a proximal function $\textsc {Proxy}$ on $\mathbf {w}$. The *i*th component of $\textsc {Proxy} (\mathbf {w}, \lambda )$ is given by the following expression:

4 $$\begin{aligned} \textsc {Proxy} ((\mathbf {w},\lambda )_i):= {\left\{ \begin{array}{ll} w_i - \lambda , &{} \text{ if } w_i > \lambda , \\ 0, &{} \text{ if } |w_i| \le \lambda , \text{ and } \\ w_i + \lambda , &{} \text{ if } \ w_i < -\lambda . \end{array}\right. } \end{aligned}$$

*Step size* $\eta$. An important parameter when using Gradient Descent is the size of the steps. If the step size is too small, the algorithm will need too many iterations, while if it is too large, the algorithm will never converge. In Algorithm 1, the step size decreases in every iteration, such that the weight vector converges. The initial step-size is typically a user-chosen parameter; however, if it depends on the input data, then it needs to be calculated securely. For example, one could choose $\eta _0 = \frac{0.1}{\max (\mathbf {X}^t \mathbf {X})}$, of which we explain the secure calculation in section "Secure lasso regression".

*Goodness of fit.* To test the performance of Lasso Regression we can use different goodness of fit measures, such as the mean squared error, the mean absolute error or the coefficient of determination $R^2$. As an example for the secure implementation, we focused on the last measure. $R^2$ provides a measure of how well observed outcomes are replicated by our prediction model, based on the proportion of total variation of outcomes explained by the model. The range of $R^2$ is $[-\infty ,1]$, where 1 indicates the best fit. We calculate $R^2$ as follows. First, we define the value $\bar{y}$ as the mean of the observed target data $y_i$, i.e. $\bar{y} = \frac{1}{n} \sum _{i=1}^n y_i$. We then denote by $\mathbf {y}^{\text {pred}}$ the vector of the values predicted by the model, and define

$$\begin{aligned} R^2 = 1 - \frac{\sum _{i=1}^n (y_i - y_i^{\text {pred}})}{\sum _{i=1}^n(y_i - \bar{y})}. \end{aligned}$$

This coefficient is important for determining the right regularization parameter $\lambda$. In practice, one will have to run the Lasso regression multiple times with different $\lambda$, to find the optimal model with the highest $R^2$.

### Description of the secure solution

#### Aim and assumptions

The goal of this section is to show how the functionality described in "Description of the desired functionality" section  can be realized in a *secure* way: this means that while both parties will learn the output of the Lasso regression (i.e. the model coefficients) trained on the inner join of their datasets,^4^ no other information on the datasets of each party will be disclosed to any other party.

Our secure solution involves a third party, which does not supply any input, and does not receive any output (except from the size of the intersection of the two datasets). For our Proof of Concept, this third-party role is taken by ZorgTTP, a company that offers consultancy and practical solutions on the topic of privacy-preserving data analysis in the healthcare sector. The addition of such a party has two benefits, relating to the two steps of our solution: secure inner join and secure Lasso regression. For the first step, the presence of a third party allows us to design a custom, highly efficient protocol; for the second step, we are able to use the MPyC library [35], that provides useful building blocks but requires at least three parties to guarantee security.

Before discussing the details of our solution, we give a brief introduction to Secure Multi-Party Computation. Notice that we chose to present cryptographic concepts with a focus on intuition, so as not to burden the reader with an unnecessary level of formalism. The reader can refer to [36, 37] for a more formal discussion of general cryptographic concepts (including cryptographic hash functions, homomorphic encryption, and secret sharing), and to [38, 39] for an in-depth discussion of MPC and Secret Sharing.

#### Introduction to secure multi-party computation

Assume *n* parties $P_1, \ldots ,P_n$ hold private inputs $x_1, \ldots ,x_n$. An MPC protocol is an interactive protocol that allows the parties to compute the value $f(x_1, \ldots ,x_n)$ of a function *f* on their inputs, without revealing any other information to each other on their inputs. Notice that the private inputs are not necessarily just a single element, but could actually consist of an entire dataset. Moreover, not all parties need to supply an input, and not all parties should receive an output; the addition of these “data-less” parties (such as ZorgTTP in our case) allows protocols to achieve better efficiency, or to use techniques which would be insecure with a smaller number of parties.

#### Secure inner join

As outlined in section "Inner join of the data", in order to realize a protocol that securely implements our desired functionality, the first step to be performed is to compute the so-called inner join of the datasets of Achmea and Erasmus MC. Namely, we need to obtain a database with the identifiers that are present in both the datasets of Achmea (AC) and Erasmus MC (EMC), and with the corresponding features coming from both datasets. Notice that we do *not* wish to reveal the dataset obtained in this way to any party, as it would still contain highly sensitive personal data (in case of application involving real data). The inner-join database will thus remain secret — yet computing the coefficients of a Lasso regression model on this secret dataset will be possible.

We first give a brief overview of the cryptographic building blocks that are used for this phase, and then present our solution.

*Cryptographic Building Blocks.* Our solution makes use of three core components: *(keyed) cryptographic hash functions,* *(additively) homomorphic encryption,* and *2-out-of-2 secret sharing.*

- *Hash functions.* A cryptographic hash function is a deterministic function $H:\mathcal {D}\rightarrow \mathcal {C}$, that maps any alphanumeric string $\mathbf {s}\in \mathcal {D}$, to another alphanumeric string $H(\mathbf {s})=\mathbf {z}\in \mathcal {C}$, called *digest,* of fixed length^5^ Such a function enjoys the property that, given a digest $\mathbf {z}\in \mathcal {C}$, it is unfeasible to compute a string $\mathbf {s}$ such that $H(\mathbf {s})=\mathbf {z}$. In our protocol, we compute the hash of values $\mathbf {s}$ that are concatenated with a random bit-string $\mathbf {b}$, thus obtaining $H(\mathbf {b}\Vert \mathbf {s})$. This ensures that a party with no knowledge of $\mathbf {b}$ is unable to recover $\mathbf {s}$ from its hash with a brute-force attack; in cryptographic terms, it is a simple form of keyed hashing.
- *Homomorphic encryption.* An (additively) homomorphic encryption scheme is a public-key encryption scheme, thus consisting of a key-generation algorithm $\texttt {KeyGen}$, an encryption algorithm $\texttt {Enc}$ and a decryption algorithm $\texttt {Dec}$. For a keypair of public and secret key $(\text {pk},\text {sk})$ generated by $\texttt {KeyGen}$, we have that $\texttt {Enc}_{\text {pk}}$ takes as input a message *m* and some randomness *r*, and produces as output a ciphertext $c=\texttt {Enc}_{\text {pk}}(m,r)$, with the property that $\texttt {Dec}_{\text {sk}}(c)=m$, and that no information whatsoever can be extracted on *m* or $\text {sk}$ from *c* and $\text {pk}$; in formal terms, the encryption scheme is IND-CCA1 secure. In order to simplify notation, we will often omit the key and randomness when discussing encryption, and write $\left[ m \right] :=\texttt {Enc}_{\text {pk}}(m,r)$; moreover, we implicitly assume messages to be numeric values, so that addition and subtraction of messages are well-defined. The scheme is supposed to be additively-homomorphic, which means that there exists special operations on ciphertexts $\boxplus$ and $\boxminus$, such that $\left[ m_1\right] \boxplus \left[ m_2\right] = \left[ m_1 + m_2\right]$, and $\left[ m_1\right] \boxminus \left[ m_2\right] = \left[ m_1 - m_2\right]$ for all messages $m_1, m_2$.
- *2-out-of-2 secret sharing.* This building block can be seen as a form of key-less encryption, distributed among two parties, and works as follows: given a secret (numerical) value *s*, two elements $s_1$ and $s_2$ called *shares* are randomly sampled, but subject to the condition that $s_1 + s_2 =s$. Then $s_1$ is assigned to a party, and $s_2$ to another party; in this way, each party has individually no knowledge of *s* (since the share $s_i$ that they have is a random number), but the original secret value *s* can be reconstructed, when the two parties cooperate and communicate their shares to each other.

*The Secure Inner Join Solution.* The presence of a third party (ZorgTTP) allows us to design a novel, highly efficient protocol for secure inner join, which we believe to be of independent interest. ZorgTTP is taking care of the communication between the two data holders, but is not allowed to learn any data other than the cardinality of the intersection. The goal is for AC and EMC to obtain a secret-shared version of the features from Table 5. Our secure inner join protocol between AC, EMC, and ZorgTTP uses *cryptographic hash functions*, and both AC and EMC have an *(additively) homomorphic encryption* key pair; we used SHA-256 [40] as hash function and the Paillier homomorphic-encryption scheme [41] in our implementation. Both public keys are communicated to the other involved parties, so that everyone can encrypt and perform homomorphic operations on ciphertexts; denote by $\left[ x \right] _{\text {AC}}$ a value encrypted under the public key of AC, and by $\left[ x \right] _{\text {EMC}}$ a value encrypted under the public key of EMC. The main idea is as follows:

1. AC and EMC randomly permute the rows of their datasets.
2. AC and EMC jointly generate a random bit string, not known to ZorgTTP, for the cryptographic hash function.
3. Using the hash function and the random string, both AC and EMC hide the identifiers from their own databases, and send the obtained hashes to ZorgTTP. AC and EMC then use their own public key to homomorphically encrypt the feature values of their records, and send the resulting ciphertexts to ZorgTTP. For simplicity, we assume here that AC and EMC have only one feature each. More features can be processed by a simple repetition of the steps below; the reader can refer to Table 6 below for a visual representation of the data sent to ZorgTTP, and to “Appendix” section for the details.
4. ZorgTTP computes how many hashed identifiers from AC also appear among the hashed identifiers of EMC; denote by $k$ this value. Due to the properties of cryptographic hash functions, $k$ is equal to the number of records in the intersection of the datasets of AC and EMC, and ZorgTTP learns no other information on the identifiers. For simplicity, we assume $k=1$. In case $k$ is larger, the steps below can be repeated, ZorgTTP properly linking the attributes with overlapping hashed identifiers; once again a more detailed description can be found in “Appendix” section. Note that, at this point, ZorgTTP holds the encrypted features of this record, which we denote by $\left[ \alpha \right] _{\text {AC}}$ and $\left[ \beta \right] _{\text {EMC}}$.
5. Both AC and EMC generate a random value, denoted by *s* and *z* respectively.
6. Both AC and EMC use the public key of the other party to homomorphically encrypt their shares, thus obtaining $\left[ s \right] _{\text {EMC}}$ and $\left[ z \right] _{\text {AC}}$, and they then send these ciphertexts to ZorgTTP.
7. ZorgTTP computes $\left[ \alpha \right] _{\text {AC}} \boxminus \left[ z \right] _{\text {AC}} = \left[ \alpha -z \right] _{\text {AC}}$ and sends this to EMC. Similarly, ZorgTTP computes and sends $\left[ \beta -s \right] _{\text {EMC}}$ to AC. Table 7 below visualized the data obtained and computed by ZorgTTP.
8. AC and EMC decrypt the received values and obtain $\beta -s$ and $\alpha -s$, respectively. Note that we have thus obtained a 2-out-of-2 sharing of $\alpha$ and $\beta$ among EMC and AC, since AC still holds *s* and EMC still holds *z*. This outcome can be seen in Table 8.

To ensure that the decrypted differences (in step 9) reveal no information on the feature values, the randomly generated shares (in step 6) need to be sufficiently large.

**Table 6:** Encrypted data sent to ZorgTTP by AC and EMC, respectively

Hashed identifier	Encrypted feature $\alpha$	Hashed identifier	Encrypted feature $\beta$
$H(a_1\Vert r)$	$\left[ \alpha _1 \right] _{\text {AC}}$	$H(b_1\Vert r)$	$\left[ \beta _1 \right] _{\text {EMC}}$
$H(a_2\Vert r)$	$\left[ \alpha _2 \right] _{\text {AC}}$	$H(b_2\Vert r)$	$\left[ \beta _2 \right] _{\text {EMC}}$
$\vdots$	$\vdots$	$\vdots$	$\vdots$

**Table 7:** Encrypted data obtained and intersected by ZorgTTP

Matching identifiers	Feature $\alpha$	Feature $\beta$	Value AC	Value EMC
$H(a_i\Vert r) = H(b_j\Vert r)$	$\left[ \alpha \right] _{\text {AC}}$	$\left[ \beta \right] _{\text {EMC}}$	$\left[ \alpha -z \right] _{\text {AC}}$	$\left[ \beta -s \right] _{\text {EMC}}$

**Table 8:** Final tables of secret-shares obtained by AC and EMC, respectively

$\alpha$-share	$\beta$-share	$\alpha$-share	$\beta$-share
$\alpha -z$	*s*	*z*	$\beta -s$

#### Secure lasso regression

Once the steps of Paragraph "Secure inner join" section have been performed, we obtain a “2-out-of-2 secret-shared” version of Table 5: namely, Achmea and Erasmus MC each have a table filled with apparently random numbers, but if they were to add up the corresponding numbers, they would obtain exactly Table 5.

Recall that our purpose is to train a linear regression model — specifically, Lasso — on this table. Now letting Achmea and Erasmus MC communicate their datasets to each other in order to reconstruct Table 5, and then train the regression model, is clearly *not* an option: the information that they would obtain consists of personal data, the exchange of which has to be prevented.

Instead, we present a solution that is able to compute the regression coefficients from the two datasets, *without* leaking information on their content.

The fundamental building block that allows us to design and implement this solution is Shamir Secret Sharing. We make use of the software platform MPyC [35], which implements this form of secret-sharing, and other useful communication and computation tools. We present the relevant properties and features of MPyC and Shamir Secret Sharing in the next section, and then discuss how these are used in our solution.

*Shamir Secret Sharing* As mentioned above, the core component of MPyC is Secret Sharing due to Shamir [42]. Shamir Secret Sharing can still be seen as a form of key-less distributed encryption, but the number of involved parties and the privacy and reconstruction guarantees are different. Instead of discussing Shamir Secret Sharing in its full generality, we focus here on the regime of parameters which is relevant for our purposes, called (1, 3)-*Secret Sharing.*

Given three parties $P_1, P_2, P_3$, a (1, 3)-secret-sharing scheme (denoted by SSS for short) consists of two algorithms, namely a *sharing* $\texttt {Share}$ and a *reconstruction* algorithm $\texttt {Rec}$. $\texttt {Share}$ is, in general, randomized, and on input a given (secret) value *s*, it outputs three elements $\mathbf {s}_1, \mathbf {s}_2, \mathbf {s}_3$ called shares. Typically, any party can use the sharing algorithm to obtain shares of a secret value of their knowledge, and they will then distribute these shares to the parties, with party $P_i$ receiving share $s_i$. The $\texttt {Rec}$ algorithm tries to invert the process: on input three elements $\mathbf {s}_1, \mathbf {s}_2, \mathbf {s}_3$, it outputs a value *s* or an error message $\bot$, indicating that the reconstruction failed.

A (1, 3)-SSS enjoys 1-*privacy:* no information on the secret value *s* can be extracted from an individual share $s_i$. On the other hand, two or more shares allow to unequivocally reconstruct *s* (2-*reconstruction*), i.e. $\texttt {Rec}(s_{i_1},s_{i_2})=s$.^6^ The $``1''$ in (1, 3)-SSS thus refers to the privacy threshold, while the $``3''$ refers to the total number of parties.

Such a secret-sharing scheme can be used to construct MPC protocols: assume that the three involved parties (Achmea, Erasmus MC, and ZorgTTP) have access to a (1, 3)-SSS. Let us assume that parties wish to perform some computation on a value $\alpha$ (held by Achmea) and $\beta$ (held by Erasmus MC). The three parties can then proceed as follows: first, Achmea secret-shares $\alpha$, i.e. computes $(\alpha _1,\alpha _2,\alpha _3)=\texttt {Share}(\alpha )$, such that Achmea, Erasmus MC and ZorgTTP will receive $\alpha _1, \alpha _2, \alpha _3$, respectively. Notice that by 1-privacy, no information on $\alpha$ is leaked at this point. Erasmus MC then similarly secret-shares $\beta$, i.e. computes and distributes $(\beta _1,\beta _2,\beta _3)=\texttt {Share}(\beta )$.

The key property now is that for any operation that the parties wish to perform on the values $\alpha$ and $\beta$, there exists a corresponding operation that can be performed *on the shares* $\alpha _i,\beta _i$, resulting in some other sharing $s_1,s_2,s_3$, in such a way that no information at all is leaked on $\alpha$, nor $\beta$. It is important to remark that these operations typically involve all shares and may also require some form of communication among the three parties. While operations such as sum can be straightforwardly be evaluated, multiplications are typically more involved; MPyC makes use of a relatively standard protocol where players locally multiply shares, then re-share the obtained values and apply a Lagrange interpolation function on the received shares [43, 44].

It then becomes possible to evaluate a complex algorithm such as Lasso regression on several features of Achmea and Erasmus MC: parties can secret-share their features, then decompose the Lasso regression into basic operations, and perform the corresponding operations on the shares. Eventually, they will obtain shares of the regression coefficients; due to the 2-reconstruction property, Achmea and Erasmus MC at this point simply need to exchange their shares with each other and to evaluate $\texttt {Rec}$ in order to obtain the coefficients.

A final remark of notable importance is that while sums and multiplications are, per se, sufficient to evaluate any algorithm, MPyC also supports a number of custom sub-protocols to evaluate special operations in a much more efficient way. Notably, efficient systems are implemented to compute the maximum of two values and to evaluate the inner product of two vectors, and there is full support for fixed-point arithmetic operations; we refer to the protocol specifications [35] for the details.

*Casting from 2-out-of-2 to Shamir Secret Sharing.* Recall that once the steps in section "Secure inner join" have been executed, parties obtain a 2-out-of-2 secret sharing of the table which serves as input for the secure Lasso solution, and not the (1,3) secret sharing that is required for MPyC. The first step to be performed is thus to “cast” this 2-out-of-2 secret sharing to a (1,3)-Shamir sharing.

This is actually a fairly simple step, where only sum operations are required. Indeed, denote by *x* a 2-out-of-2 share of Achmea and by *y* the corresponding 2-out-of-2 share of Erasmus MC, which means that $x+y=z$, where *z* is some feature value of an individual record occuring in both datasets. Now Achmea can (1,3)-share *x* and Erasmus MC can (1,3)-share *y*, so that Achmea obtains $x_1$ and $y_1$, Erasmus MC obtains $x_2$ and $y_2$, and ZorgTTP obtains $x_3$ and $y_3$. All parties have now have to locally add their shares, resulting in $x_1+y_1$, $x_2+y_2$, and $x_3+y_3$: these are valid (1,3)-shares of $z=x+y$ that can be used in MPyC.

*The Secure Lasso Regression solution.* In order to explain our secure Lasso solution, we follow the blueprint of section "Lasso regression algorithm" and show how each step can be securely performed on secret shared data, using the techniques of section "Secure lasso regression".

- *Secure Gradient Descent.* Apart from the stopping criterion, $\textsc {CalcGradient}$, $\textsc {Proxy}$, step size $\eta$ and goodness of fit $R^2$, all computations in Algorithm 1 are linear operations, and can thus be calculated on secret-shared data as explained in section "Secure lasso regression". We will now elaborate on these secure calculations.
- *Secure stopping criteria.* As explained in the corresponding paragraph in section "Gradient descent approach", there are two possible stopping criteria: The first one is reaching the maximal number of iterations, and since $\text {maxIter}$ is a public value, this criterion does not need to be implemented securely: The second criterion demands computing the update difference $\textsc {UpdateDifference}$, and compare this with the tolerance (which is a public constant). For efficiency purposes, we chose not to implement all these steps securely. Instead, for every iteration we reveal the value of the update difference, and compare it with the tolerance in plaintext. To be more precise, recall that the update difference is given by the ratio between $||\mathbf {w}_{\text {new}}-\mathbf {w}_{\text {old}}||_2^2$ and $||\mathbf {w}_{\text {old}}||_2^2$: in order to calculate the update difference, we securely compute both enumerator and denominator and then reveal their values. We believe the information leak of this step to be acceptable, especially given the performance gain that is derived from it by avoiding the expensive secure division step.
- *Secure*$\textsc {CalcGradient}$*and*$\textsc {Proxy}$. In order to securely calculate the gradient of $\mathbf {w}$, linear operations are used. We also make use of the custom sub-protocol for vector multiplication, as described in section "Secure lasso regression". In order to compute $\textsc {Proxy}$, we calculate two secret-shared bits, namely $a = \left( w_i > \lambda \right)$, and $b = \left( w_i < - \lambda \right)$, where $(x<y)$ denotes the bit that is equal to 1, if $x<y$, and to 0, otherwise. We can then securely compute the following linear operation over the shares of $w_i$:
  5 $$\begin{aligned} \textsc {Proxy} (w_i) = a\cdot (w_i - \lambda ) + b \cdot ( w_i + \lambda ). \end{aligned}$$
  It is easy to see that this gives the same result as Eq. (4).
- *Secure initial step size.* Although the operations that we use for computing our choice of the initial step size $\eta _0$ (inner product and maximum) are computationally expensive, we only need to perform them once. Once again, we make use of the sub-protocols for vector multiplications and maximum from MPyC.
- *Secure goodness of fit.* Once we have computed the weight vector of the prediction model, we aim to securely compute goodness-of-fit measures. As an example we implemented $R^2$. Recall that the definition of $R^2$ is given by
  $$\begin{aligned} R^2 = 1 - \frac{\sum _{i=1}^n (y_i - y_i^{\text {pred}})}{\sum _{i=1}^n(y_i - \bar{y})}. \end{aligned}$$
  With the shares of $\mathbf {X}$, and the publicly-known coefficients of $\mathbf {w}$, we can calculate the shares of $\mathbf {y}^{\text {pred}}$. At this point, by using the secret-shared vector $\mathbf {y}$, we can compute the numerator and denominator of $1-R^2$, reveal these values, and thus obtain $R^2$.

## Results

In this section we first present the security results of our solution. We then discuss the scalability results of our Proof of Concept, which was not performed on real data but did run on the actual infrastructure between Achmea, Erasmus MC and ZorgTTP. Finally, we describe the performance of our implementation of the Lasso regression and the accuracy of the secure model.

### Security results

The security of our solution is guaranteed under the following assumptions. First of all, we assume that any two parties are connected by secure channels; in practice this is done by means of SSL/TLS connections. We assume that parties do follow the instructions of the protocol; in cryptographic lexicon, they are thus assumed to be *semi-honest.* Privacy is guaranteed, even if parties try to infer extra information from the data they sent and received as part of the protocol, though we assume that no party will collude with any other party and exchange information with them. Finally, we adopt the standard assumption that the involved parties are bounded by polynomial-time computations, and that factoring large integers is feasible under this constraint.

Under the above conditions, the solution we present is provably secure, in the sense that we can mathematically argue that the only information that will be revealed are regression coefficients, and the size of the intersection between the datasets of Achmea and Erasmus MC.

### Running time

We implemented our solution in Python. In order to test the efficiency of our implementation, we ran several experiments on three machines, under the control of Achmea, Erasmus MC and ZorgTTP, respectively, and geographically separated.

The experiments include the secure inner-join computation and the protocol to securely train a Lasso regression algorithm as described in section "Description of the secure solution". Notice that we have not evaluated the efficiency of applying the Lasso model to new data, as it would be out-of-scope for this article;^7^ For the same reason, no test data is extracted from these data artificial datasets.

All three (virtual) machines run a Linux-based operating system, and are equipped with a commercial-grade virtual processor (up to four cores at 2.4GHz) and with 8 to 16 GB of RAM.

The solution was installed as a Docker image on all three machines. Connections within the machines were realized via HTTPs over TCP (for the secure inner join) and via the custom TCP protocol of MPyC. The connections were secured with TLS; certificates were created and installed on the machines to this end.

In order to test the efficiency of our solution, we sampled artificial datasets, using scikit-learn (with the datasets.sample_generator.make_regression functionality, that creates a dataset of real numbers with a rougly linear dependency of the target features). We sampled datasets with an increasing number of records and features, and ran several instances of our solution. The number of records (per dataset) was equal to 5, 100, 500, 1000, 5000 and 10000, while the (total) number of features was equal to 1, 2, 5, 10, 30 and 40. We vertically split the dataset into two datasets, with an (up to one difference) equal number of features and with a complete overlap in record IDs, i.e. the identifiers in the Achmea dataset were identical to those of the Erasmus MC dataset for each iteration.

For datasets with five records, we chose not to run instances with more than two features, as this regime of parameters would be highly unsuitable for a linear regression algorithm. Furthermore, the instance with 10.000 records and 40 features could not be run due to the RAM limitations of the involved machines; we nevertheless believe that the instances we considered are sufficient to analyze the scalability of our solution.

Each instance was run 10 times; all figures presented in this article refer to the median time over these 10 executions.

**Fig. 1:**
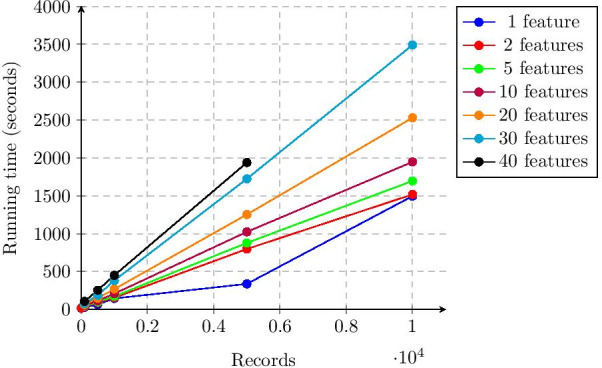
Total running time of the experiments as a function of the number of records (median values)

**Fig. 2:**
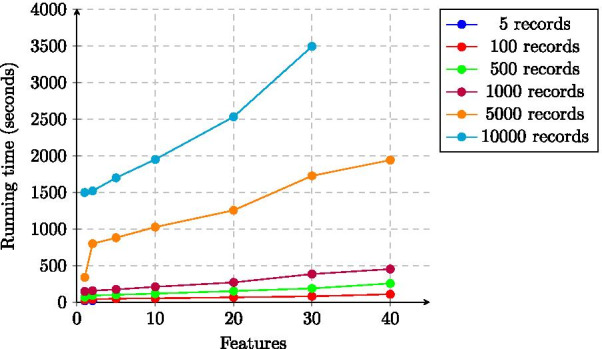
Total running time of the experiments as a function of the number of features (median values)

The total running time (thus encompassing both secure inner join and secure Lasso regression) is showed in in Figs. 1 and 2. Our solution thus takes roughly 3500 s, slightly less than one hour, to process two datasets with 5000 records each and a total of 30 features. Moreover, the running time of our solution scales linearly in the number of records and features.

**Fig. 3:**
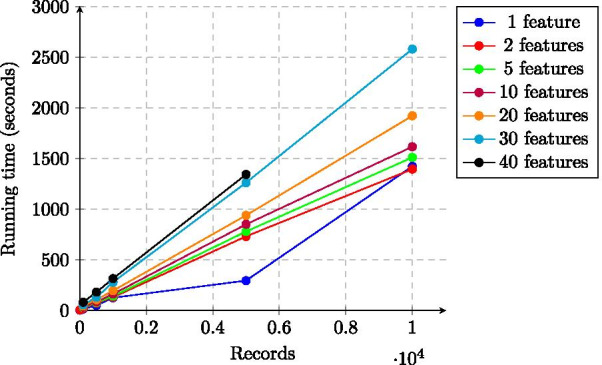
Running time of Lasso regression (training phase) of the experiments as a function of the number of records (median values)

**Fig. 4:**
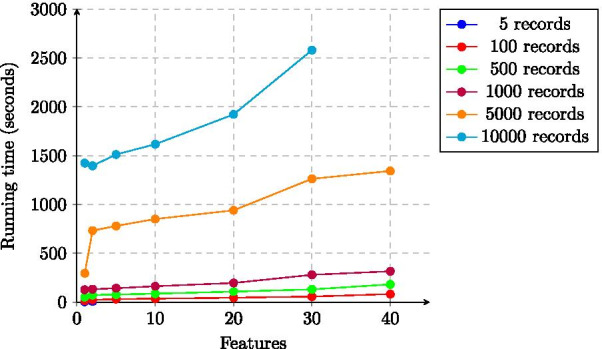
Running time of Lasso regression (training phase) of the experiments as a function of the number of features (median values)

The running time of our solution is dominated by the Secure Lasso regression, the scalability of which is shown in Figs. 3 and 4. Just as for the total time, the running time of this phase also has a linear dependency on the number of records and of features.

### Performance and accuracy results

### Data

To test the performance and accuracy of our secure model, we use the “Medical Costs” dataset by Brett Lantz [45]. This public dataset contains 1338 records of patients with 12 features each (including, among others, age, bmi, children, gender, medical costs), of which four are numerical, and eight are Boolean. We centered and scaled the data in advance, such that the feature values are comprised between 0 and 1. We also split into a train and a test set (10% of the data, randomly selected).

#### Performance of lasso regression

To test the performance of our solution we compare the results of our secure model with the non-secure scikit-learn Lasso model [46]. Note that the secure inner join has no influence on the performance of the Lasso regression. Therefore, as input of our secure model, the data is secret-shared between the three parties. The influence of the calculation on secret-shared values will be discussed in the next paragraph.

We train with our secure model on 11 features of the train set for predicting the (numerical) target feature of medical costs, by varying $\lambda$ and tolerance. We found the optimal choice, leading to a good fit ($R^2$, mean squared error) and enough coefficients set to zero, to be $\lambda = 0.001$ and tolerance $=0.0001$. For this choice of parameters, when training our secure model, we need 26 iterations. Applying the trained model on our test set, we achieve an $R^2$ of 0.70, a mean squared error of 0.0086, a mean absolute error of 0.062 and an objective of 0.013. As a validation of the solving method that we used, we compare these results with the (highly optimized) Lasso model of scikit-learn [46], using the same parameters. After the model was trained on the train set, on the test set we find an $R^2$ value of 0.66, a mean squared error of 0.012, a mean absolute error of 0.082 and an objective of 0.0090. Although the goodness-of-fit measures of our secure model are better than the scikit-learn model, it has a larger objective value. In Tables 9 and 10 one can see that in the scikit-learn model, two more coefficients are set to zero, which is one of the aims of Lasso. Therefore, we can conclude that our secure model has a good performance, although the (highly optimized) scikit-learn model performs slightly better.

**Table 9:** Comparison plaintext model and Sklearn Lasso: objective, $R^2$, mean squared error and mean absolute error

Model	Obj	$R^2$	MSE	MAE	intercept
scikit-learn	0.0090	0.66	0.012	0.082	0.39
our secure model	0.013	0.74	0.008	0.062	0.18
Abs. diff.	0.004	0.08	0.004	0.020	0.21

**Table 10:** Comparison plaintext model and Sklearn Lasso: coefficients

Model	c1	c2	c3	c4	c5	c6	c7	c8	c9	c10	c11
scikit-learn	0.08	0.01	0	0	0	− 0.30	0	0	0	0	0
our secure model	0.17	0.10	0.001	0	0	− 0.19	0.18	0	0	0	0
Abs. diff.	0.09	0.09	0.001	0	0	0.11	0.18	0	0	0	0

#### Accuracy of the secure implementation

To compare the performance and accuracy of our secure model, we implemented the Lasso algorithm described in section "Gradient descent approach" in a non-secure way. Although the steps in training both models are the same, a slight difference in outcome is to be expected, due to possible rounding errors of non-integer, secret-shared values. This difference in objective values is less than $10^{-7}$; we consider this to be negligible for our research purposes.

## Discussion

In light of the results shown in section "Results", we conclude that our solution does provide a viable way of securely training a Lasso regression model on distributed patient data in a privacy-preserving way. In particular, the good quality of the obtained model, together with its satisfying efficiency in a fairly realistic set-up, make our solution a promising tool for privacy-preserving analysis of distributed patient data.

As future work, we have identified two main directions, namely, improvements to the solution and working towards a pilot on real heart-failure risk data.

### Improvement to the secure solution

We identify several ways to further improve our solution. First of all, our solution was relatively efficient, the secure solution took less than one hour for the setting with 5000 records and 30 features. This should be fast enough for research purposes. However, while we deem our solution to be fast enough for research purposes, its efficiency might need to be improved when working with very large datasets. Several approaches are possible in order to reduce running time, for instance implementing the solution in another programming language such as C, or making optimal use of parallelisation. Moreover, RAM usage could be reduced by supporting access to advanced database-management systems.

Also, we identified some opportunities to improve the quality of the model. Within this article, we assumed the data to be pre-processed, i.e. scaled and centered; a solution with a higher technology readiness level would need to securely implement this step. Moreover, next to $R^2$, more goodness-of-fit measures such as mean squared error and mean absolute error could be securely implemented. This would enable parties to perform more quality checks on the model, and to choose a good regularization parameter $\lambda$.

Finally, while we focused on a situation where exactly two parties supply input data, and it would be interesting to extend our solution to more than two data-parties. The secure Lasso regression training poses no issue for such an extension, since MPyC supports a virtually unlimited number of parties, but the secure inner join would need to be re-designed, since it is tailored to the two-party-with-helper setting. A step-by-step approach for this part could probably be realized, i.e. by first performing an inner join of the datasets of two parties and then using the outcome as input for another inner join with the third data party, and so on, but a thorough analysis is required to validate this approach and measure its performance.

### Towards prediction of heart-failure risk factors

Given the promising results obtained by our Proof of Concept, a future pilot with real patient data should be started, in order to establish the effectiveness of our solution for prediction of heart-failure risk on combined datasets from Erasmus MC and Achmea. The data needed for such an experiment is already stored at both parties. At Achmea, features express and quantify, notably, the number of days a given customer was admitted to a hospital, and various other aspects such as comorbidities, marital status, and socio-economic status. This information is stored as part of the standard procedures of health insurance companies. At Erasmus MC, on the other hand, features express and quantify social and behavioral aspects such as age, smoking, exercising, and alcohol consumption. This type of data has been collected by the Epidemiology department of Erasmus MC as part of a previous study performed on volunteers in the city of Rotterdam [3], of which a significant part are also ensured at Achmea. In a future pilot, we would aim to predict the number of hospitalization days as a function of the other feature values. Such a pilot would need to address both the technical challenges highlighted above (for instance, Achmea has data on more than five million individuals). But it should also focus on non-technical challenges, such as compliance and legal aspects, and ensure that employees and management are properly involved in the process and get acquainted with the used techniques, which constitutes a time-consuming process.

## Conclusions

In this paper, we presented a secure and scalable solution for Lasso regression as a part of the European BigMedilytics project. The solution allows two parties, in this case Erasmus MC and Achmea, to securely compute the inner join of their respective datasets and to train a Lasso regression algorithm on the obtained dataset in a privacy-preserving way, assisted by healthcare information intermediation company ZorgTTP. No party learns any patient data, other than the number of overlapping patients from both datasets, the result of the regression, and some intermediate values of the regression algorithm, which we believe to be fully acceptable.

We implemented our solution on three computing nodes, running at separate machines, and located at different sites, under control of Achmea, Erasmus MC, and ZorgTTP, respectively. The experimental results show that our implementation is reliable, accurate, and fast enough for research purposes. We conclude that our solution is a promising tool for privacy-preserving machine learning tasks on distributed patient data, potentially leading to an improvement of the quality of healthcare, while respecting the privacy of involved patients.

### Supplementary information

**Additional file 1:** Zip archive containing the log files of the scalability experiments. The archive can be opened with any archive manager, and the included log files (with .log extension) can be read with any text editor.

## Data Availability

The (artificial) datasets used for the scalability experiments are included in the supplementary material, in the $\texttt {artificial\_datasets\_scalability.zip}$ file. The log files measuring the running time of the solution for these inputs datasets are also included, to be found in $\texttt {log\_files\_scalability.zip}$. Finally, the dataset used to test the accuracy of our solution is a publicly-available dataset [45].

## Appendix: Secure inner join protocol

This is a detailed version of the secure inner join protocol, described in “Aim and assumptions” section. Both AC and EMC get a key pair of an additively homomorphic crypto system, which keys are generated in the first step. In the second step a random key *r* is generated jointly between AC and EMC, without ZorgTTP learning it. This key is used for scrambling the identifiers and encrypting the private data in step 3. They are sent to ZorgTTP in step 4, together with the encrypted attribute values. In step 5 ZorgTTP looks for the matching indices of the scrambled identifiers, and obtains the intersection cardinality. In steps 6 and 7, AC and EMC generate their shares of the inner join table entries, and send them encrypted to ZorgTTP. In step 8, ZorgTTP computes the encrypted remaining shares, so AC and EMC can decrypt them.

## Footnotes

1 https://gdpr-info.eu/.

2 https://www.govinfo.gov/content/pkg/PLAW-104publ191/pdf/PLAW-104publ191.pdf.

3 https://www.bigmedilytics.eu/.

4 To be completely precise, we also reveal the size of the intersection of the two datasets to the involved parties.

5 Domain and codomain do not need, strictly speaking, to consist of alphanumeric strings, but we restrict to this situation for simplicity.

6 Formally, each share $s_i$ should be supplied to $\texttt {Rec}$ together with its index *i*, but we omit this to simplify notation.

7 In the healthcare-scenario that motivates this article, applying the model could arguably be performed without advanced cryptographic techniques, since the smaller amount of data needed would probably make direct data usage feasible and proportional (under appropriate informed consent). For this reason, we have decided not to focus on the application of the trained Lasso model.
